# Belling the Cat

**Published:** 1908-11

**Authors:** 


### BELLING THE CAT.

**A BLOODLESS WAR.** **AN EDITORIAL ROAST**

The Association of Medical Editors is a brave lot, I don't think. It is composed principally, if not entirely, of editors of what are generally understood to be the independent medical journals; but just where the independence comes in it is somewhat difficult to see; seeing that they have not had the independence to cut loose from the Octopus and hit back. It is true they are "privately owned and run for profit," as Jonesy-the-Joke, says; that is, they are not subject to orders of the Octopus, as are its tentacles, the State Association journals.

But, when it is realized that the principal and evident and declared aim of the Octopus and its satellites is to *kill them off,* choke them off, starve them to death, by any and every means—foul or fair—war, open and secret, dictation, bull-dozing, intimidation, misrepresentation, it looks to me *cowardly* not to hit back; not even to resent the most flagrant and outrageous insult that could have been offered them. I refer to the public pillorying and proscription of the entire class, individually and collectively, of journals carrying uncensored advertisements that was published by the Octopus and its feeders prior to a recent meeting of the A. M. A. at Atlantic City: Here it is:

"No medical journal or publication can be exhibited that contains advertisements of drug or chemical or similar preparation used in the treatment of disease which does not conform to the rules of the Council on Pharmacy and Chemistry of the American Medical Association."

Here is a companion piece to it, from the *Texas State Journal of Medicine,* March, 1907:

"These journals might do a useful work. We wish them well, but they must clean house or be labeled 'infectious' and be shunned by the profession."

I reproduce some of my comments on this, at the time, May, 1907:

The enforcement of this ukase will exclude practically every medical journal published in America and Canada, except the "State" journals—those wearing the collar of the Octopus and bearing the union stamp.

Are we living in America, or in darkest Russia? Is this the fourteenth or twentieth century? Have we—the owners and publishers of legitimate medical journals—any rights whatever, that the Machine is bound to respect? Have we the right to think and speak our sentiments, or are we to be controlled by the monstrous machine which would "muzzle the press" under the denunciation of being "unclean," "infectious" and unworthy of support by the profession? Shall we not be permitted to "differ with Brother Paul" as to the respectability of a pharmacal preparation? Who gave this council the right to dictate what the press shall and shall not advertise? This is the most insolent, high-handed and outrageous act of all the brazen acts of the political machine that controls the A. M. A. and its organ, the Octopus. Will the effrontery and arbitrary exercise of a self-assumed authority on the part of the Octopus stop here? Hardly. If this is submitted to by the American medical editors—those who do not wear the collar and the union stamp—we may look, next, to see an inspector with the A. M. A. union badge stationed at each door at future meetings to search our persons to see if any one has concealed about him a copy of any of the "infectious" and "unclean" publications. We may look to see such person arrested, caught in the act of smuggling contraband publications, and arraigned on a charge of treason to the autocrat—the Czar of all "schools of medicine." Shall we, like Socrates for "corrupting the youth of Athens," be made to quaff the hemlock for "corrupting" the younger men of medicine by insisting that there is virtue in pharmacy-by-the-large, as well as by the obsolete prescription compounding; and that thousands of the most experienced physicians use—and find useful—hosts of the preparations advertised in the medical press? Will our editorial utterances be censored next? The Association of Medical Editors will meet at Atlantic City, June 4th, simultaneously with the A. M. A. We will see what they will do about this monstrous assumption of authority. If they tamely submit to the thraldom—submit to being put in irons, as it were, and exhibited in the pillory as "unclean" and "infectious," and unfit to be represented in the A. M. A.—they may next expect to be bodily kicked out, and they would deserve to be! These editors are all members of the A. M. A. and pay tribute to Cæsar. ("Red Back," May, 1907.)

Well, they did nothing about it. They "tamely submitted." They swallowed the insult. They had not the manliness to resent it and withdraw individually and as a body from affiliation with their assailants. True, Coe did make a feeble kick, but was whipped into line and apologized.

Now, the war on the independents (God save the mark) is to be waged warmer than ever. The tentacles have organized an association of their edtiors, including Jonesy-the-Joke, who was refused membership in the A. M. E. Association (took their spite out on poor Jonesy), and the screws are to be tightened. I have a suspicion that the drastic order of the Postmaster General's Department compelling all of the journals to drop delinquents was instigated by the Octopus combine. This is illegitimate warfare; it is like the Chinese throwing stink pots.

But, seeing that its warfare upon the independent medical press up to date has been bloodless—with a single exception—the aggressor is trying new tactics. That exception was the little *Medical Gazette* of Fort Worth, whose obituary we wrote at the time, and which I reproduce here, as germane to the subject.

ELEGY IN A JOURNAL CHURCHYARD.

"The Octopus tolls the knell of smaller fries;
Its wily tentacles creep slowly t'wards the sea;
The *Gazette* turns up its little toes and dies,
And leaves the field to Matthew [Smith] and to me."

"THE RAID OF THE BRAVE HOME GUARD."

In fact, the war by the Octopus upon the "independent press" reminds me of Captain Wilborn's "brave home guards" (during the Civil War), when they raided, and the little burlesque song the boys sang in commemoration of it.

"Little Captain Wilborn of Mississip,
Made a raid into Alabam;
Killed three black snakes, captured a gopher,
Without the loss of a single man."

Now, you may ask: what has all this to do with belling the cat? Why, the American Medical Editors take it out in talking. They meet and abuse the Octopus, in prose and rhyme; denounce it, yet do nothing. Like the rats, they made fun of the cat (behind its back), but listened for and trembled at its stealthy tread. They resolved that that cat should wear a bell, so that they could hear it coming, and *hunt their holes*. But—who shall bell the Octopus cat? Search *me!*

---

THE SALOON.—The archenemy of the human family. The one evil of our times that overshadows all others, that is more far-reaching and deadly in its evil influences than all others. It stands for the wail of unhappy women and the cry of hungry children. It means widows and orphans; ruins health and homes. It stands for crime, insanity, poverty, shame, sickness, sorrow, death! The workingmen, its victims, chiefly, who squander their wages in "setting' 'em up" for the saloon bums, when his wife and children are hungry, when his home—if he have one—is mortgaged, resents any interference with his "personal rights" to spend his wages, and says that the saloon keeper will lend him a dollar when he needs it [sure!]. Poor fools! They have not intelligence enough to realize that enlightened men are their best friends, and would save them from themselves, and their families from misery and ruin, and they regard all such as enemies. May God help them! The saloon must go. It is the duty of every physician to preach the gospel of temperance, fresh air and cleanliness within and without. Selah!

---

THE TUBERCULOSIS CRUSADE.—So far as I have been able to gather from the journal and newspaper reports of the International Congress on Tuberculosis, the time was spent in threshing over old straw; a reiteration of well known facts; an academic discussion of the disease, rather than a practical application of the principle of sanitation for its arrest—and prevention. I mean—the result, so far as practical dealing with the subject goes—may be summed up in two words: *nihil fit*. True, much new matter was discussed, and interest seemed to center around the question raised by Koch, whether the disease can be transmitted from the cow to man. We all know how extensive the prevalence of the disease is; how fatal; what relation its mortality bears to that of other diseases; what the money loss to the country is; and all that; and we know, practically, how it is engendered in unsanitary conditions of life and labor—how it is communicated from the sick to the well. So, what's the use of the endless iteration of these things? Why not *do* something? It was expected, at least, that the Congress would urge the creation of a Department of Public Health, with a head to administer and an organization to execute the details of a practical application of sanitary science for the prophylaxis of the disease, and the indiscriminate dissemination of the infection. It should be dealt with as in any other infectious disease; the first step of such dealing should be—report of all cases to the proper authorities.

But, if the Congress did nothing else but talk, influences were put in motion that will no doubt lead to practical results. Each State will probably take up the work, and institute measures for prevention of the spread. The first step Texas took, and Texas is the first to act—as usual—was to organize a strong league. See communication in this issue from Secretary Bibb. The first thing this league should do should be to notify railroads and other transporters that they must not bring any consumptives into Texas, invoking the law on the subject, which fixes a heavy penalty for its violation. Dr. Daniel said in an address before the American International Congress at St. Louis in October, 1904: "The days for talking are past, and the time for action has come. We are here to do something. * * * The purposes for which we are met are to put in motion influences that will awaken a public sentiment and lead to legislation; to induce Congress and the States to pass measures and enforce them; to lessen the fearful mortality of this disease; and ultimately to do what may be done toward its eradication. * * * We do not know all about consumption, but we know how it is caused, how propagated, how communicated from the sick to the well, and how it may be prevented. Let us go ahead and prevent it.

"But it requires authority and means to devise and enforce those measures, and the lawmakers alone can give that authority. We are here, then, for that purpose; to interest the public and the lawmaking bodies; to show them these things; to bring them to a realizing sense of the useles sand perpetual waste of life by a preventable disease. In short, to set in motion influences that will lead to efficient action by the Federal government."

That meeting gave the initial impulse to the movement now so general. It is useless to talk of "educating the masses by pamphlets and tracts." The under stratum—the toilers and sweaters—wouldn't, couldn't read them. If they could, they couldn't understand. If they did, they couldn't act. They are as children, and government must stand in *loco parentis* to them; must think and act for them. John Mitchell voiced the cry of the helpless in his address before the Washington Congress. He voiced my sentiments also: "For God's sake do something." "We are ignorant of the danger and powerless against it." It was the most sensible address during the Congress. I reproduce it here:

THE CRY OF THE VICTIMS.—JOHN MITCHELL BEFORE THE GREAT CONGRESS ON TUBERCULOSIS.

Mr. Mitchell said:

"To the men of learning and science who have gathered in this Capital City from all parts of the globe, the working people of America turn with expectancy and confidence. We men of labor who carry more than our full share of the burdens and make our full share of sacrifices, cry aloud for assistance and direction in our struggle against this terrible plague, which, unaided, we can not successfully combat.

"In common with all other factors in society, we are alive to the importance of researches made to find a cure for those already afflicted, but we are equally—even more—concerned in regard to measures which will prevent infection and stop forever the spread of this disease.

"Immunity from the infection and relief from those things which predispose workingmen and women to consumption must be brought to us in the places in which we live and work. It is, of course, a source of gratification to know those more favored by fortune, who are victims of this disease, may find relief in other climes, but the men and women of toil are compelled by circumstances beyond their control to remain not only in the community where they contracted the disease, but often are obliged to continue in their employment until they succumb to its ravages.

"Among the obstacles to greater progress in the promotion of health and the eradication of disease is the attitude of many employers of labor. Not only do they resist the enactment of laws for the prevention of accidents and the promotion of health, but it is with the greatest reluctance that they comply with the laws which have been enacted.

"Equally important are the housing conditions in our large cities. Owners of property feel little responsibility for the health or comfort of tenants; their chief concern seems to be to secure the largest possible return upon their investments, and all attempts to enforce by law regulations of a sanitary character are denounced as unnecessary and unwarrantable interference.

"It would be, of course, unjust to say that employers or landlords should be blamed for all the evils which affect and threaten the lives of the working people. There is much that the wage earners may do to alleviate conditions to prevent the dissemination of disease germs and to cure tuberculosis in its incipient stages. The danger may be minimized by the application of simple and natural rules of life and conduct; and to the extent that education and agitation may offer relief, it is the duty of the working people to take advantage of the practical suggestions which are made for their guidance and to follow the advice given for their benefit. Cleanliness, fresh air and temperate living are the best preventives of disease, and in most cases these essential requisites are within reach or control of the working people. But, unfortunately, either through negligence or carelessness, or both, the simplest and most obvious rules of health are disregarded with the result that the grave claims countless victims of an easily preventable disease.

"It is eminently fitting that one session of this great International Congress should be given to the consideration of tuberculosis in its relation to the wage earners, and it is safe to predict that much valuable information and many good results will come from the addresses of the distinguished gentlemen who have honored us with their presence."

---

An Epistle to My Delinquents.—A true physician is, essentially, a gentleman. A gentleman has a high sense of honor. He discharges all obligations, and pecuniary obligations are essentially matters of honor. It is hard to understand how any reputable physician can reconcile it to his self-respect and the crudest conception of honor to take, read and enjoy a publication for which he has subscribed and never countermanded, month after month, year after year, and utterly ignore every appeal made to him for settlement. And it is almost incredible that there are, in Texas, physicians of high standing who will ignore a subscription bill of a few dollars, sent him four times a year for two, three, four, five years. Attention has been called time and again to the ruling of the Postoffice Department requiring publishers to drop all such delinquents. But still there are many from whom I have not heard. Surely no self-respecting physician whose subscription is in arrears will allow me to be forced to cut him off, so long as he is in debt to me. It is a matter of honor. I have no discretion. I need the money. I want the friendship and support of these men, and I hope this, last appeal, will bring a prompt response.

